# Chemometric Analysis of Cannabinoids: Chemotaxonomy and Domestication Syndrome

**DOI:** 10.1038/s41598-018-31120-2

**Published:** 2018-08-30

**Authors:** E. M. Mudge, S. J. Murch, P. N. Brown

**Affiliations:** 1 Chemistry, University of British Columbia, Kelowna, British Columbia Canada; 2 Natural Health & Food Products Research, British Columbia Institute of Technology, Burnaby, British Columbia Canada; 3 Biology, University of British Columbia, Kelowna, British Columbia Canada

## Abstract

Cannabis is an interesting domesticated crop with a long history of cultivation and use. Strains have been selected through informal breeding programs with undisclosed parentage and criteria. The term “strain” refers to minor morphological differences and grower branding rather than distinct cultivated varieties. We hypothesized that strains sold by different licensed producers are chemotaxonomically indistinguishable and that the commercial practice of identifying strains by the ratio of total THC and CBD is insufficient to account for the reported human health outcomes. We used targeted metabolomics to analyze 11 known cannabinoids and an untargeted metabolomics approach to identify 21 unknown cannabinoids. Five clusters of chemotaxonomically indistinguishable strains were identified from the 33 commercial products. Only 3 of the clusters produce CBDA in significant quantities while the other 2 clusters redirect metabolic resources toward the THCA production pathways. Six unknown metabolites were unique to CBD-rich strains and/or correlated to CBDA and 3 unknowns were found only in THC-rich strains. Together, these data indicate the domestication of the cannabis germplasm has resulted in a loss of the CBDA pathway in some strains and reallocation of resources between CBDA and THCA pathways in others. The impact of domestication is a lack of chemical diversity and loss of biodiversity in modern cannabis strains.

## Introduction

*Cannabis sativa* L. (marijuana) is a dioecious, annual plant from Central Asia that has been used medicinally and recreationally for thousands of years^1^. The domestication of cannabis has included human selection, inbreeding and cross breeding as well as natural outcrossing and genome mixing^1^. Strains are not easily delineated by genotype and only moderate correlations have been observed between *C. indica* and *C. sativa* ancestry. In addition, large genetic variance has been observed within identically named strains^2,3^. Standardized, highly controlled programs to breed elite varieties or cultivars by selection of phytochemical profile have been limited^4,5^. It is estimated that there are several hundred or perhaps thousands of strains of cannabis currently being cultivated in legal and illegal markets^4^. It is possible that chemically identical or very closely related plant material is being sold under several different names by different producers and there is no clear definition of the concept of a “strain”.

Cannabis producers market their products based on the amounts of total Δ9-tetrahydrocannabinol (THC) and cannabidiol (CBD) with the assumption that the overall phytochemical composition of the material can be extrapolated from these values, but there is considerable anecdotal evidence suggesting that strains with similar THC/CBD content have different effects on human physiology^6,7^. More than 120 different cannabinoids have been described in cannabis^8,9^ with the most interesting phytochemistry found in the glandular trichomes on the flowers of the female inflorescences^10^. THC is the most researched cannabinoid and there are ten additional classes of cannabinoids with varying chemical structures^8^. Cannabinoids are synthesized in acidic forms through the condensation of geranyl diphosphate (GPP) and most commonly olivetolic acid, products of the methylerythritol phosphate (MEP) and polyketide pathways^11,12^. There are several other polyketides that can be used in place of olivetolic acid, which contribute to the wide variation within this chemical class^13,14^. Neutral cannabinoids are products of decarboxylation from processing and handling harvested flowers.

Chemometric models are used to evaluate metabolite datasets to delineate relationships and identify potential influences on phytochemical diversity^15–17^. These approaches can be classified as targeted analysis, untargeted phytochemical discovery, metabolomic profiling or fingerprinting^15^. Targeted metabolomics determines differences in known phytochemicals while the untargeted approaches evaluate unidentified compounds in the phytochemical profiles^15^. Targeted-untargeted approaches combine known metabolites with the untargeted datasets as a hypothesis-generating tool to discover metabolite relationships, clusters, families and biochemical pathways^15,18^. The use of these models and algorithms enables a better understanding of metabolite commonality and diversity within plant species^19^.

We hypothesized that the total THC and CBD content is not sufficient to distinguish strains and that a combination of targeted and untargeted chemometric approaches can be used to predict cannabinoid composition and to better understand the impact of informal breeding program and selection on the phytochemical diversity of cannabis. To investigate these hypotheses, we assembled a collection of cannabis strains sold by licensed producers in Canada primarily based on total THC/CBD content, and analyzed the strains for known cannabinoids using a previously validated analytical method^20^ to establish clusters of similar plant materials.

We then used an untargeted metabolomics approach to identify previously uncharacterized compounds and chemical relationships. We identified 5 clusters of chemotaxonomically indistinguishable strains within the collection. Our results show that the variation in less abundant cannabinoids between cannabis strains was not dependent on the total THC and CBD content. These data suggest that the domestication of the cannabis germplasm has resulted in the loss of the CBDA pathway in some strains and the reallocation of resources between CBDA and THCA pathways in others.

## Results

### Targeted Metabolomics of Cannabinoids

**Figure 1:**
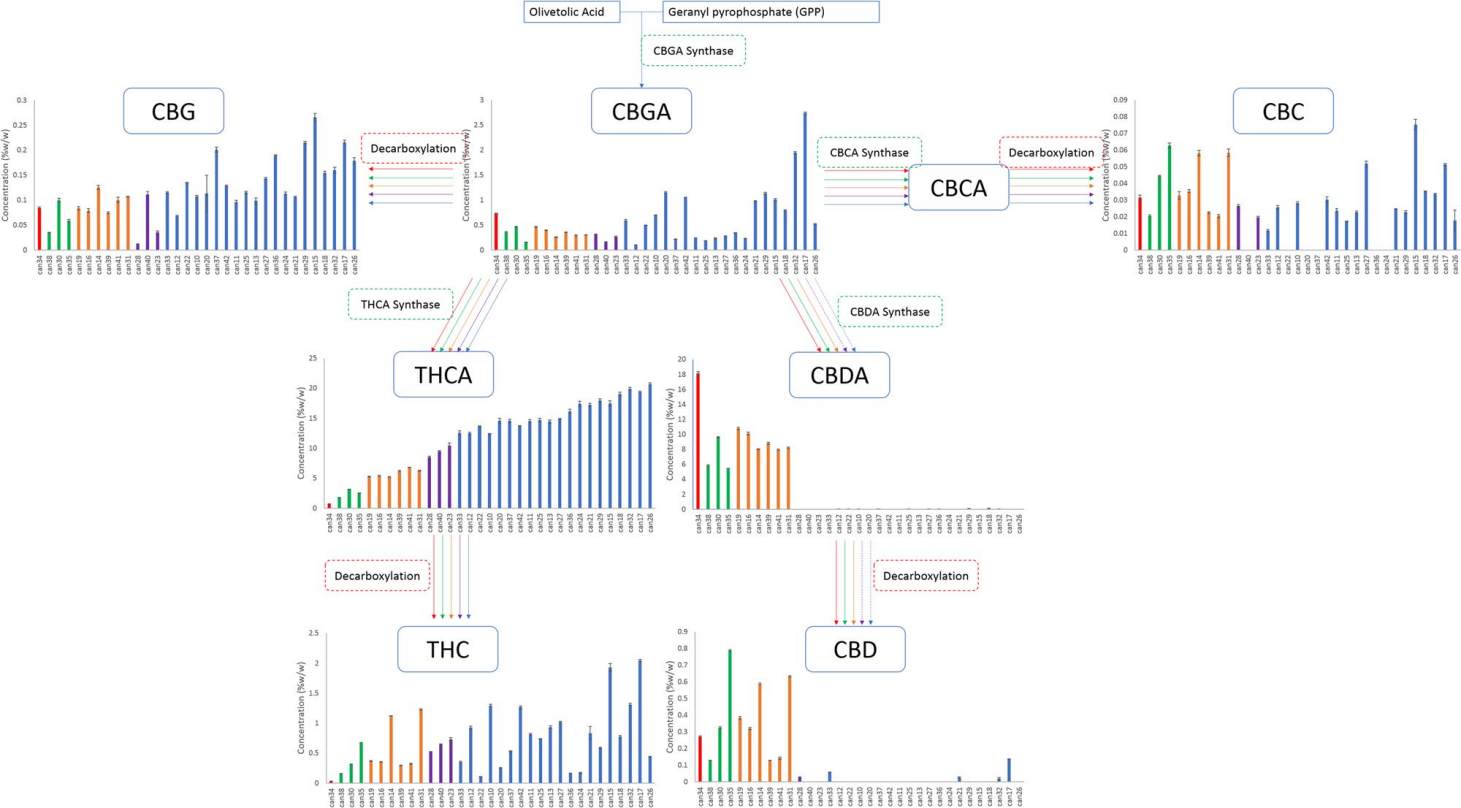
Biosynthetic pathway of cannabinoids originating from olivetolic acid and geranyl pyrophosphate. Graphs describe the cannabinoid contents within the 33 strains obtained arranged from lowest to highest total THC.

Two cannabinoids for which standards were obtained, CDBV and CBL, were not detected in any strain. The 11 remaining cannabinoids with available chemical reference standards were identified and quantified. THCA content ranged from 0.76 to 20.71% w/w, with almost a linear increase in content from the lowest to highest strain with an r^2^ of 0.97, while CBDA content ranged from <MDL to 18.11% w/w, with the highest CBDA strains having the lowest THCA contents (Fig. 1). In THC abundant strains the CBDA levels were less than 0.15%, while in CBD abundant strains the content was greater than 5%. THC, the decarboxylated form of THCA was present in strains from <LOQ up to 2% by weight in some strains, while CBD contents ranged from <MDL to 0.8%. CBD was most prevalent in high CBDA strains. In addition, 7 cannabinoids present at lower levels were quantified using individual calibration standards: THCV, CBG, CBN, CBC, CDBVA, CBGA and Δ8-THC.

### Classification of Strains

We hypothesized that individual plant breeders selected for cannabis strains by up-regulating and down-regulating specific enzymes within the biosynthetic pathways resulting in a redirection of metabolites between THCA and CBDA. Our data analysis identified 5 clusters of strains that fall within a narrow range of total CBD/THC values consistent with this hypothesis (Table 1). The branch of the biosynthetic pathway with olivetolic acid and geranyl pyrophosphate as precursors produces CBGA, CBG, CBCA, CBC, THCA, THC, CBDA and CBD (Fig. 1). Strains from all clusters contained measurable amounts of CBGA, CBG, THCA and THC (Fig. 1). Nine strains from the clusters with higher concentrations of THCA (blue and purple) did not contain detectable levels of CBC (Fig. 1). Two of the clusters were not found to contain significant quantities of CBDA and CBD (Fig. 1; blue and purple). One strain was different from all others and had a greater CBDA content and detectable levels of CBGA, CBG CBC, and CBD with minimal THCA and THC (Fig. 1; red).

**Table 1:** Strains of cannabis were clustered into 5 distinct groups that could be separated by the flow of metabolites through the CBD and THC pathways.

Group	Colour Code	CBD Range (% w/w)	THC Range (% w/w)	# Strains
A	Blue	<MDL–0.08	11.3–19.1	20
B	Purple	<MDL–0.02	8.0–9.9	3
C	Orange	7.1–9.7	5.0–6.7	6
D	Green	5.3–8.8	1.7–3.1	3
E	Red	16.1	0.7	1

**Figure 2:**
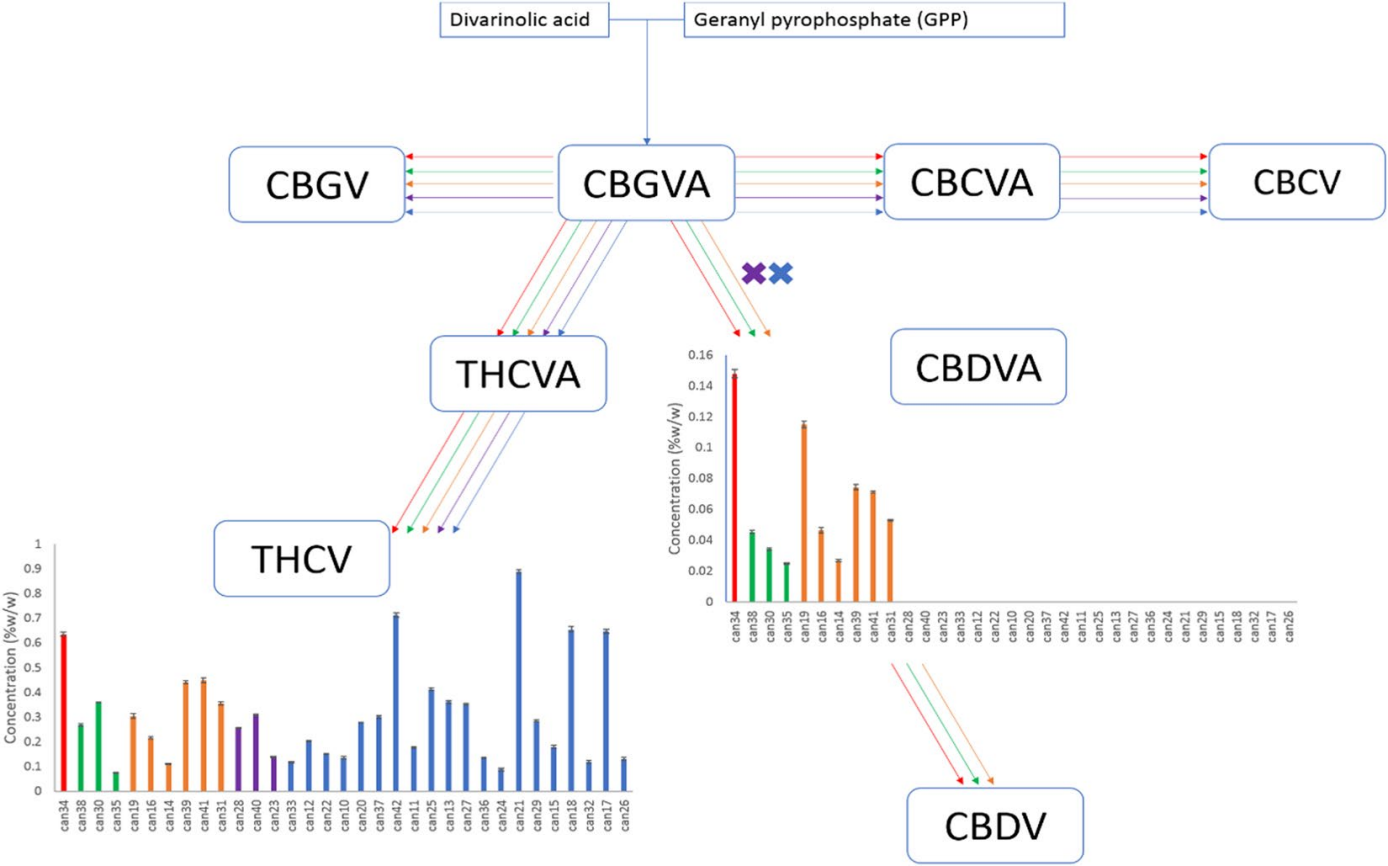
Biosynthetic pathway of cannabinoids originating from divarinolic acid and geranyl pyrophosphate. Graphs describe the cannabinoid contents within the 33 strains obtained arranged from lowest to highest total THC.

Compounds produced from the precursors divarinolic acid and geranyl pyrophosphate via CBGVA were also found to differ by strain cluster (Fig. 2). CBGVA appears to be a branch point for allocation of resources in cannabis between THCV and CBDVA indicating that the enzyme activity or the resource allocation mechanism for production of THCV was lost in the breeding process of strains clustered in the red, orange and green groups (Fig. 2).

### Untargeted Metabolomics Analysis

In addition to the 11 cannabinoids that corresponded with authentic standards, 21 peaks were identified in the chromatograms with UV spectra characteristic of cannabinoids. By comparison to THC, the contents were estimated from <MDL up to 0.34% by weight. Two unknown cannabinoids (CMPD-7 and CMPD-11) were detected in all strains, while CMPD-3 and CMPD-20 were each only detected in a single strain.

### Relationships Between Known and Unknown Cannabinoids

**Figure 3:**
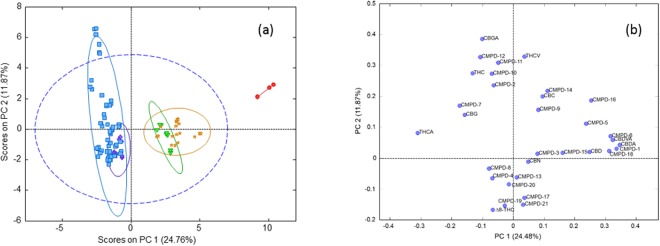
Principal Component Analysis (PCA) of cannabinoid profiles classified according to THC/CBD contents (**a**) scores plot (**b**) loadings plot.

A principal component analysis (PCA) of the autoscaled cannabinoid data was plotted to show the clustering of the samples in an unsupervised fashion (Fig. 3). In the PCA plot, the first two principal components (PC) captured 36.6% of the variance in the data. Based on the loadings plot, the first PC was most highly influenced by the THCA and CBDA content of the strains, which are negatively correlated. There are two high THC strains (CAN17 and CAN21) and one CBD strain (CAN34) that were separated from the data clustered within the 95% confidence limit of the total data variance. Based on the loadings plot (Fig. 3B), CAN17 and CAN21 may be influenced by a significant number of low abundance cannabinoids including CBGA, CMPD-12, and CMPD-11. CAN34 is likely due to its significantly higher CBDA content relative to the other strains and because it contained less than 1% total THC.

While the first two principal components of PCA describe 36% of the variance, there is a remaining 64% of the variance in the cannabinoids not being described with this model. Therefore, additional models were employed to understand the relationships between cannabinoids and to identify additional strain classes based on the content of these 32 different cannabinoids. Multiple linear regression (MLR) analysis showed that 14 cannabinoids were better suited compared to all cannabinoids for predicting THCA content with validation r^2^ values improving from 0.02 and 0.88, respectively and for predicting CBDA content 14 cannabinoids improved the validation r^2^ values from 0.49 to 0.95 when compared with using the entire data set.

Pearson correlations were used to determine whether any of the unidentified cannabinoids could be associated with the major cannabinoids THCA, THC, CBDA and CBD (Table 2). There was no significant correlation of THCA or THC and any of the unknown compounds (Table 2). The CBDA content was positively correlated with CMPD1, CBDVA, CMPD5, CMPD6. CMPD16 and CMPD18 (Table 2). CBD was potentially weakly correlated with CMPD1, CMPD6 and CBDA (Table 2).

**Table 2:** Pearson correlation coefficients of all cannabinoids relative to the four major cannabinoids (THCA, CBDA, THC and CBD) in addition to UV spectral analysis describing cannabinoids as acidic or neutral.

Cannabinoid	UV Spectrum: Acidic/Neutral	THCA	CBDA	THC	CBD	Putative ID
CMPD1	Acidic	−0.71	0.93	−0.39	0.54	CBDA-C1
CMPD2	Acidic	0.22	−0.14	0.03	−0.11	
CBDVA	Acidic	−0.70	0.93	−0.36	0.49	
CMPD3	Acidic	−0.20	0.20	0.15	0.40	
CMPD4	Acidic	0.18	−0.19	0.17	−0.20	
CMPD5		−0.36	0.61	−0.26	0.12	
CMPD6	Acidic	−0.65	0.84	−0.27	0.58	CBDA-C4
CBDA	Acidic	−0.81	1.00	−0.34	0.68	
CMPD7	Acidic	0.53	−0.41	0.21	−0.29	
CMPD8	Acidic	0.21	−0.26	0.12	0.01	
CBGA	Acidic	0.46	−0.18	0.52	−0.17	
CMPD9		−0.29	0.22	0.23	0.30	
CMPD10	Neutral	0.16	−0.12	0.35	−0.12	
THCV	Neutral	0.05	0.16	0.15	−0.10	
CMPD11	Neutral	0.36	−0.09	0.31	−0.21	
CBD	Neutral	−0.68	0.68	0.00	1.00	
CMPD12	Neutral	0.28	−0.23	0.24	−0.12	
CBG	Neutral	0.66	−0.35	0.43	−0.27	
CMPD13	Neutral	−0.04	0.02	0.23	0.17	
CMPD14	Neutral	−0.19	0.34	0.07	0.20	
CMPD15	Acidic	−0.24	0.39	−0.32	0.08	CBDMA
CMPD16	Neutral	−0.55	0.68	−0.08	0.44	
CMPD17		0.00	0.19	−0.19	0.07	
THCA	Acidic	1.00	−0.81	0.39	−0.68	
CBN	Neutral	−0.26	0.32	−0.05	0.41	
CMPD18	Neutral	−0.73	0.91	−0.25	0.72	CBDM
THC	Neutral	0.39	−0.34	1.00	0.00	
8-THC	Neutral	0.28	−0.14	−0.13	−0.07	
CBC	Neutral	−0.21	0.30	0.63	0.59	
CMPD19	Neutral	0.19	−0.08	−0.24	−0.21	
CMPD20	Acidic	−0.04	−0.11	0.01	−0.10	
CMPD21	Neutral	0.02	0.05	−0.05	0.18	

### Putative Identifications and Pathways

**Figure 4:**
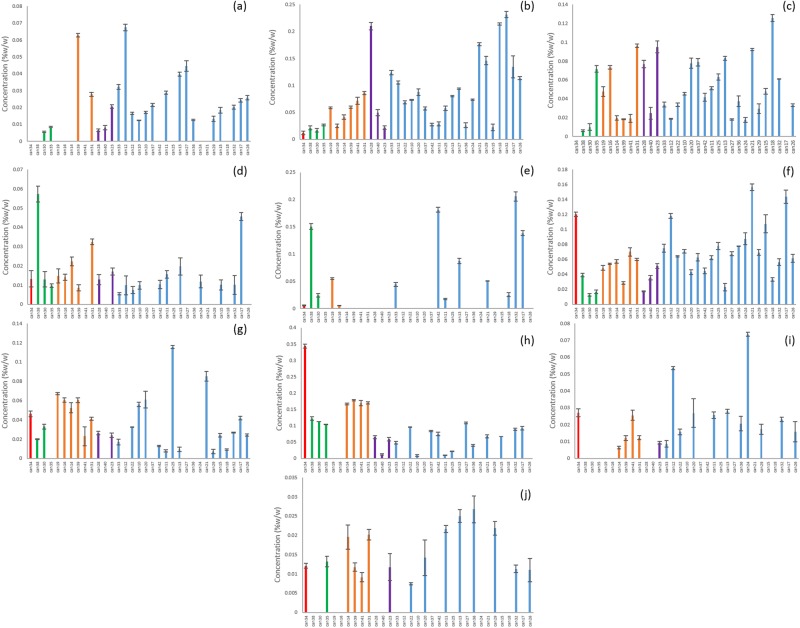
Unknown cannabinoids determined by untargeted metabolomics analysis to be common to all clusters of strains. (**a**) CMPD4, (**b**) CMPD7, (**c**) CMPD8, (**d**) CMPD9, (**e**) CMPD10, (**f**) CMPD11, (**g**) CMPD14, (**h**) CMPD16, (**i**) CMPD19, (**j**) CMPD21.

**Figure 5:**
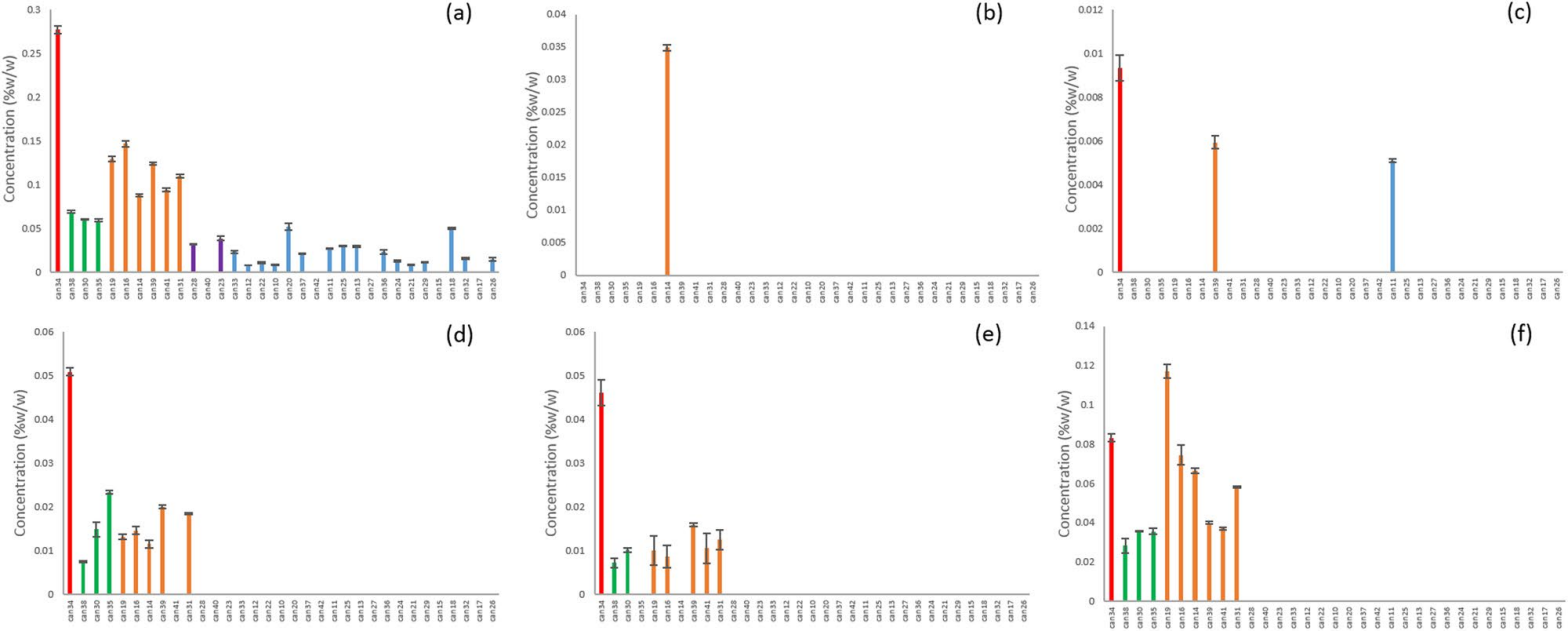
Unidentified cannabinoids determined by untargeted metabolomics analysis to be unique to CBD-rich strains. (**a**) CMPD1, (**b**) CMPD3, (**c**) CMPD5, (**d**) CMPD6, (**e**) CMPD15, (**f**) CMPD18.

**Figure 6:**
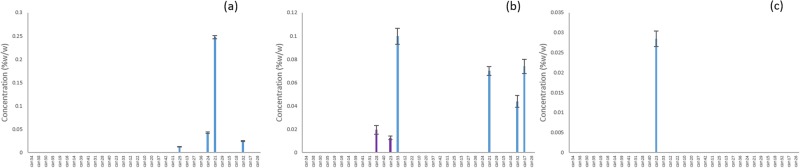
Unidentified cannabinoids determined by untargeted metabolomics analysis to be unique to THC dominant strains. (**a**) CMPD2, (**b**) CMPD12, (**c**) CMPD20.

Ten of the unknowns were found across multiple strains from all of the clusters (Fig. 4). CMPD1 was strongly correlated with CBDA according to Pearson’s correlation (Table 2) and although it was found in many of the strains classified as blue or purple, it was at much higher concentrations in the red, green and orange clusters (Fig. 5a). Compounds 3,5,6,15 and 18 were found only in the CBD-rich clusters red, green and orange (Fig. 5b–f). Compounds 2, 12, and 20 were found only in THC dominant strains (Fig. 6a–c).

## Discussion

The long history of human use has made the exact region of origin for cannabis difficult to establish, though literature supports Northeast Asia^1,4,21^. Breeding of cannabis cultivars in the hemp industry has focused on morphological improvements through established breeding programs, while marijuana, or drug-type cannabis, has primarily taken place in underground/clandestine programs through crossing landraces and/or “indica” and “sativa” lineages^22,23^. The major focus of breeding was increasing the yield of THC, although other features were considered including organoleptics (aroma), morphology, color and trichome density^1,4,24^. The genetic diversity between marijuana strains is lower in comparison with hemp varieties due to crossing closely related varieties^2,3^. CBDA and THCA synthases are thought to be controlled by two alleles on a single locus (B), crossing of CBDA and THCA dominant strains will produce offspring with intermediate total THC:CBD ratios^5,25^. With the prevalence of propagation through cuttings of mother plants, feminization of seeds and production of sensimilla, the need for male plants has decreased resulting in potential loss of genetic and phytochemical diversity^1^. The “sativa” and “indica” lineages used to describe cannabis throughout the industry are based on postulation that sativa strains originated from European hemp cultivars, while indica are from potent, resinous Indian cannabis^4^ but given the use and trade of the plant in ancient times, the exact origin is unknown and these may not be distinct species^21^. Modern strains are considered dominant in either of these two “lineages” or hybrids between close relatives. These classifications focus on the pharmacological effects associated with the strains where sativa plants are considered stimulating and indica plants are associated with relaxation and sedation but this is not a botanical or chemotaxonomical classification^23^. Comparisons of cannabinoid contents of these classifications have shown that the THC content can be identical between these two classification groups^3,26^. Many questions remain: What is a “strain”? Does a “strain” represent a phytochemically unique variety? Are “strains” from different growers actually different? Is there a more appropriate way to classify “strains”? Are these cultivars, varietals, landraces or even species? What is the impact of domestication on the ecological fitness of the species?

Breeding closely related plants potentially leads to loss of genetic diversity within the genome^27^. Traits signifying domestication syndrome include phenotypic changes such as increase seed size, loss of shattering, changes in reproduction, changes in secondary metabolites and loss of pest resistance compared with wild ancestors^27,28^. Reviews of cannabis breeding have summarized domestication in terms of morphology, while focus on secondary metabolism has focused primarily on THC content^1,4^. Recent forensic evaluations of confiscated sensimilla cannabis in the US has shown dramatic increases in total THC content over the last 30 years, from 6.3% to 11.5%^29,30^ but strains with greater than 20% total THC are available in the marketplace. This artificial increase in THCA production has resulted in the loss of CBDA synthase activity in THC dominant strains. Although crossbreeding will result in THC:CBD hybrid offspring, the loss of other biosynthetic pathways is unknown due to the non-rigorous breeding programs focusing primarily on the production of a single metabolite. Our data indicate that these breeding programs have also impacted unknown related metabolites with undetermined function.

Metabolomics analysis can generate chemotaxonomic classifications of plants in addition to hypothesis generating insight of data correlations, metabolite identification and relationships that would not be possible through single metabolite evaluation^15,31^. Using the correlation data and PCA loadings plots, we can hypothesize the putative identity of some of these unknown cannabinoids. For example, CMPD6 had a Pearson correlation coefficient of 0.89 with CBDA and occupies the same space within the PCA loadings plot. The UV spectra with a maximum of 224 nm identifies this compound as an acidic cannabinoid which was only detected in the presence of CBDA. Further evaluation showed that it eluted between CBDVA and CBDA, therefore is hypothesized to be CBDA-C4 with a butyl side chain on the polyketide (Table 2)^32^. Likewise, we putatively identified CBDA-C1, CBDM, and CBDMA among the unknowns separated by our chromatography protocol (Table 2). Due to the presence of THCA synthase in all strains, the correlation cannabinoids produced with this enzyme is less obvious. It was previously reported that low abundance cannabinoids may be regulated by upstream biosynthesis of precursor polyketides^14^. We found fewer unknown cannabinoids in the strains selected for higher THC content. With such strong emphasis on the synthesis of a single metabolite there is a strong possibility that other biosynthetic pathways have been lost in the process^27,28^.

Several classification systems have been proposed for cannabis based on a limited number of phenotypic attributes^1,4,33^. The concept of a “strain” does not reflect the crop domestication, breeding programs or plant chemistry. The strains available in the Canadian marketplace are closely related, and evaluating single metabolite classes does not provide sufficient information to understand the phytochemical diversity available. The abundance of secondary metabolites within plants does not necessarily correlate with pharmacological significance and with cannabis there is the postulated “entourage effect” describing the synergistic effects of many metabolites for anecdotal medical efficacy^7^. Domestication of the crop has limited the genetic variability in the crop and the impact on crop diversity, physiology and metabolism is not fully understood. Further research is needed to evaluate the low abundance cannabinoids for potential medicinal efficacy and to determine their roles in plant metabolism.

## Methods

### Reagents

Methanol, acetonitrile, ammonium formate and formic acid (98%) were HPLC grade. Water was deionized and purified to 18.2 MΩ using a Barnstead Smart2Pure nanopure system (Thermo Scientific). Cannabinoid standards for quantification were purchased from Cerilliant Corp. (Round Rock, TX) for tetrahydrocannabinolic acid (THCA), THC, cannabidiolic acid (CBDA), CBD, cannabigerol (CBG), cannabichromene (CBC), tetrahydrocannabivarin (THCV) and cannabinol (CBN), Δ8-THC, cannabidivarinic acid (CBDVA), cannabidivarin (CBDV), cannabigerolic acid (CBGA) and cannabicyclol (CBL). All standards were provided as 1.0 mg/mL solutions in either methanol or acetonitrile.

### Plant Materials

Thirty-three strains of cannabis were purchased from five licensed producers in Canada under the Access to Cannabis for Medical Purposes Regulations and laboratory analysis was performed under a Health Canada Controlled Drugs and Substances License. The test samples were provided as whole or milled flowers in 5, 10 and 15 gram packages and stored at room temperature until use. Due to the legal restrictions pertaining to the storage of cannabis strains, submission of voucher specimens to a herbarium were not possible, but given the regulatory framework associated with these plants, their identify has been confirmed as *Cannabis sativa* L.

### Targeted Metabolomics of Cannabinoids

The content of 13 cannabinoids was determined according to a previously validated analytical method^20^. In brief, ground cannabis flowers (0.200 g) were extracted with 25 mL of 80% methanol in a 50 mL amber centrifuge tube for 15 minutes by sonication at room temperature with vortexing every 5 minutes, followed by centrifugation at 4500 g for 5 minutes and filtration with a 0.22 µm PTFE filter. Extracts were diluted to within the calibration range using the extraction solvent and placed in the 4 °C sample holder for same-day analysis. Chromatographic separation was performed on an Agilent 1200 UHPLC with a Kinetex C18 100 mm × 3.0 mm, 1.8 µm column (Phenomenex; Torrance, CA) using gradient elution with 10 mM ammonium formate (pH 3.6) and acetonitrile. The autosampler was maintained at 4 °C and detection was at 220 nm. The peak areas for peaks with typical acidic or neutral cannabinoid UV spectra eluting between 2.5 and 14.5 minutes were collected using Chemstation software (Agilent Technologies) and known cannabinoids were identified. Known cannabinoids were quantified in % w/w against their individual calibration curves using external calibration in Excel™. The total THC content was determined as the sum of THC and THCA in addition to the total degradation products of THC: CBN and Δ8-THC, adjusted by the molar mass ratios. Total CBD content was determined as the sum of CBDA and CBD adjusted by molar mass ratios.

### Untargeted Metabolomics

Unknown cannabinoids were identified and numbered in sequential order as they appeared in the chromatogram. Unknown cannabinoids were quantified as THC equivalents using the THC calibration curves and ordered in sequential order in the chromatogram as CMPD#.

### Data Analysis

For multivariate analysis, missing values were replaced with the method detection limit (MDL) divided by 2 for each assigned cannabinoid. In the cannabinoid profiles, where the MDL has not been determined for unassigned peaks, the missing data was replaced with half of the MDL of THC. Pearson correlation coefficients to determine relationships between metabolites were calculated using the *cor* script in R. As the concentration of a given metabolite does not necessarily correlate with pharmacological activity, the data were autoscaled by mean centering and scaling to unit variance in order to give each metabolite equal weight prior to multivariate analyses. Principal component analysis (PCA) and multiple linear regression (MLR) analysis were subsequently modeled using Solo + MIA (Eigenvector Research).

## Data Availability

The datasets generated during and/or analysed during the current study are available from the corresponding author on reasonable request.
